# Genetic Variants Associated With Sudden Cardiac Death in Victims With Single Vessel Coronary Artery Disease and Left Ventricular Hypertrophy With or Without Fibrosis

**DOI:** 10.3389/fcvm.2021.755062

**Published:** 2022-01-11

**Authors:** Juha H. Vähätalo, Lauri T. A. Holmström, Katri Pylkäs, Sini Skarp, Katja Porvari, Lasse Pakanen, Kari S. Kaikkonen, Juha S. Perkiömäki, Risto Kerkelä, Heikki V. Huikuri, Robert J. Myerburg, M. Juhani Junttila

**Affiliations:** ^1^Research Unit of Internal Medicine, Medical Research Center Oulu, Oulu University Hospital, University of Oulu, Oulu, Finland; ^2^Laboratory of Cancer Genetics and Tumor Biology, Cancer and Translational Medicine Research Unit, Biocenter Oulu, University of Oulu, Oulu, Finland; ^3^Research Unit of Biomedicine and Biocenter Oulu, University of Oulu, Oulu, Finland; ^4^Department of Forensic Medicine, Research Unit of Internal Medicine, Medical Research Center Oulu, University of Oulu, Oulu, Finland; ^5^Forensic Medicine Unit, Finnish Institute for Health and Welfare, Oulu, Finland; ^6^Division of Cardiology, Miller School of Medicine, University of Miami, Miami, FL, United States; ^7^Biocenter Oulu, University of Oulu, Oulu, Finland

**Keywords:** sudden cardiac death, left ventricular hypertrophy, coronary artery disease, genetics, medicolegal autopsy

## Abstract

**Objective:** Cardiac hypertrophy with varying degrees of myocardial fibrosis is commonly associated with coronary artery disease (CAD) related sudden cardiac death (SCD), especially in young victims among whom patterns of coronary artery lesions do not entirely appear to explain the cause of SCD. Our aim was to study the genetic background of hypertrophy, with or without fibrosis, among ischemic SCD victims with single vessel CAD.

**Methods:** The study population was derived from the Fingesture study, consisting of all autopsy-verified SCDs in Northern Finland between the years 1998 and 2017 (*n* = 5,869). We carried out targeted next-generation sequencing using a panel of 174 genes associated with myocardial structure and ion channel function in 95 ischemic-SCD victims (mean age 63.6 ± 10.3 years; 88.4% males) with single-vessel CAD in the absence of previously diagnosed CAD and cardiac hypertrophy with or without myocardial fibrosis at autopsy.

**Results:** A total of 42 rare variants were detected in 43 subjects (45.3% of the study subjects). Five variants in eight subjects (8.4%) were classified as pathogenic or likely pathogenic. We observed 37 variants of uncertain significance in 39 subjects (40.6%). Variants were detected in myocardial structure protein coding genes, associated with arrhythmogenic right ventricular, dilated, hypertrophic and left ventricular non-compaction cardiomyopathies. Also, variants were detected in ryanodine receptor 2 (*RYR2*), a gene associated with both cardiomyopathies and catecholaminergic polymorphic ventricular tachycardias.

**Conclusions:** Rare variants associated with cardiomyopathies, in the absence of anatomic evidence of the specific inherited cardiomyopathies, were common findings among CAD-related SCD victims with single vessel disease and myocardial hypertrophy found at autopsies, suggesting that these variants may modulate the risk for fatal arrhythmias and SCD in ischemic disease.

## Introduction

Approximately 80% of sudden cardiac deaths (SCDs) are due to coronary artery disease (CAD) and up to one-half occur in the absence of previously diagnosed cardiac disease (1). Regardless of the cause, cardiac hypertrophy, associated with varying degrees of myocardial fibrosis, is a risk factor for SCD and is associated with increased risk for SCD in patients with or without CAD (2). Left ventricular hypertrophy (LVH) is a common finding in both ischemic and non-ischemic SCD victims at autopsy (3, 4).

Hypertension is the most common cause of LVH in patients with CAD, but LVH is also a common finding in normotensive CAD patients (5). Myocardial weight often increases along with the severity of CAD (4). Three-vessel CAD and cardiac hypertrophy increase the vulnerability to fatal arrhythmias and SCD (4). Nevertheless, some SCDs occur in victims with only single-vessel CAD and cardiac hypertrophy found at autopsy (6).

We hypothesized that a subgroup of SCD victims with less severe CAD and cardiac hypertrophy, with varying degrees of myocardial fibrosis, are associated with rare genetic variants, which could contribute to the development of ischemic heart disease, risk of fatal arrhythmias and SCD. Our aim was to study the association of rare variants in cardiac structure and function encoding genes in SCD victims with single-vessel CAD and myocardial hypertrophy detected at autopsy, in the absence of previously diagnosed CAD.

## Methods

### Study Population

The study population was derived from the Fingesture study (The Finnish Genetic Study of Arrhythmic Events; www.clinicaltrials.gov NCT02075866), consisting of 5,869 victims of autopsy-verified SCDs in Northern Finland. Medico-legal autopsies were performed between the years 1998 and 2017 in the Forensic Medicine Unit of the Finnish Institute for Health and Welfare, Oulu, Finland, and at the Department of Forensic Medicine of the University of Oulu, Oulu, Finland by experienced forensic pathologists, using contemporary guidelines for the diagnosis of cause of death. Finnish law requires medico-legal autopsies whenever the death is not caused by a known disease, when the victim was not treated by a physician during his/her last illness, or when the death has been otherwise unexpected (Act on the Inquest into the Cause of Death, 459/1973, 7th paragraph: Finnish Law). As a result of the Finnish legislation, autopsy rates are one of the highest in the Western countries (7). Deaths were defined as sudden if the event was either witnessed within 6 h of the onset of symptoms or an unwitnessed within 24 h when the victim was last seen in a stable state of health. Only sudden deaths considered to be caused by a cardiac disease were included in the Fingesture study. Non-cardiac causes of death such as pulmonary embolism, cerebral hemorrhage as well as intoxications and other non-natural causes were excluded from the SCD cohort.

### Autopsy Data

Determination of the cause of death was based on autopsy findings and complementary analyses, available medical records, police reports and specific questionnaires for the relatives of the victim. All postmortem examinations included histological examination from samples taken at autopsy. If autopsy findings were insufficient to define a cause of death, or if a toxic exposure was suspected, toxicologic investigation was conducted. In Finland, meticulous cardiac examinations are performed in all victims during medico-legal autopsies, including macroscopic investigation of myocardium and coronary arteries, heart weight and several histological samples from myocardium. Evidence of an acute coronary event, defined by the presence of an acute intracoronary thrombus, plaque rupture or erosion, intraplaque hemorrhage, critical stenosis (>75%) or complete occlusion of a main coronary artery, was used to classify SCD as ischemic. Possible myocardial scarring was detected macroscopically from cross-sectional samples of the ventricles and microscopically from the myocardial samples. A heart weight > the predicted value based on body surface area (at least 420 g) with hypertrophic myocytes observed at autopsy was identified as cardiac hypertrophy. Methods for the classification of cause of death have been described in our previous studies (3, 8).

### Tissue Samples and Gene Sequencing

DNA samples for genetic studies were isolated from formalin-fixed paraffin-embedded myocardial tissue samples taken at autopsy. For library preparation we used The TruSight Cardio gene panel kit, which contains 174 genes associated with hereditary cardiac diseases that are most affected by a genetic predisposition (http://support.illumina.com/downloads/trusight-cardio-product-files.html) (Illumina, San Diego, CA). Genes included in the panel are shown in Table 1. Purification of the samples was performed with Agencourt AMPure XP beads (Beckman Coulter Life Sciences, Indianapolis, IN). With quantitative polymerase chain reaction–based formalin-fixed paraffin-embedded quality control kit (Illumina), the quality of the samples chosen for NGS was verified. The samples that passed quality control (i.e., with a quantitative polymerase chain reaction ΔCq value ≤ 2.3) were selected for gene panel sequencing with NextSeq550 platform (Illumina). BWA Enrichment (BWA Genome Aligner Software and the GATK Variant Caller) was used to align sequences and call variants within the BaseSpace Genomics computing environment (Illumina); VariantStudio to annotate, filter, and classify the variants; and Integrative Genomics Viewer visualize the data to exclude falsely annotated variants and sequencing artifacts. Within BaseSpace, the *in silico* prediction tools PolyPhen and SIFT were used to predict the effect of amino acid changes on protein function.

**Table 1:** Cardiac structure- and function-related genes sequenced in the panel, classified by related diseases.

HCM related	*ACTC1*, ACTN2, ANKRD1, *CALR3, CAV3*, CSRP3, *JPH2*, MYBPC3, MYH6, MYH7, *MYL2, MYL3*, MYLK2, *MYO6, MYOZ2, MYPN*, NEXN, *PDLIM3, PLN, PRKAG2, TCAP, TNNC1, TNNI3, TNNT2*, TPM1, *TRIM63, VCL*
AC related	*DES*, DSC2, DSG2, DSP, JUP, *LMNA*, PKP2, *PLN*, RYR2, *SCN5A, TGFB3, TMEM43*
DCM related	ABCCP9, ACTN2, *ACTC1*, ANKRD1, *BAG3*, CRYAB, CSRP3, *DES, DMD*, DSG2, *EYA4, GATAD1*, LAMA4, LDB3, *LMNA*, MYBPC3, MYH6, MYH7, *MYPN*, NEXN, *PLN, RBM20, SCN5A, SGCD, TAZ, TCAP, TMPO, TNNC1, TNNI3, TNNT2, TPM1*, TTN, *VCL, ZBTB17*
LVNC related	DTNA, LDB3, *LMNA, MIB1*, MYBPC3, MYH7, *PRDM16, TAZ, TNNT2*, TPM1
Metabolic disorders and syndromes with cardiac diseases and congenital heart defects	*ALMS1, BRAF, CBL, COX15, CRELD1, DNAJC19, DOLK, FXN, GAA, GLA, HFE, HRAS, JAG1, KRAS, LAMP2, MAP2K1, MAP2K2, NKX2-5, NODAL, NOTCH1, NRAS, PTPN11, RAF1, SCO2, SDHA, SHOC2, SMAD4, SOS1, TBX3, TBX20, TBX5, TTR, ZIC3*
Arrhythmic disorders (LQTS, Brugada, familial FA, CPVT etc.)	*AKAP9, ANK2, CACNA1C, CACNA2D1, CACNB2, CALM1*, CASQ2, *CAV3, DPP6, GJA5, GPD1L, HCN4, KCNA5, KCND3, KCNE1, KCNE2, KCNE3, KCNH2, KCNJ2, KCNJ5, KCNJ8, KCNQ1, NPPA, RANGRF*, RYR2, *SCN1B, SCN2B, SCN3B, SCN4B, SCN5A, SNTA1, TRDN, TRPM4*
Dyslipidemia	*ABCG5, ABCG8, APOA5, APOB, APOC2, APOE, CETP, GPIHBP1, LDLR, LDLRAP1, LMF1, LPL, PCSK9, SREBF2*
Aortopathies/EDS	*ACTA2, COL3A1, COL5A1, COL5A2, EFEMP2, ELN, FBN1, FBN2, MYH11, MYLK, SLC2A10, SMAD3, TGFB2, TGFB3, TGFBR1, TGFBR2*
Muscular dystrophies/myopathies	*ACTA1, BAG3, EMD, FHL1, FKRP, FKTN, LAMA2, RYR1, SEPN1, SGCB, SGCD, SGCG, SLC25A4, TMEM43*
Other	*APOA4, CBS, CREB3L3, CTF1, FHL2, GCKR, HADHA, HSPB8, ILK, KLF10, LTBP2, MURC, PRKAR1A, SALL4, TXNRD2, ZHX3*

*Variants detected in the present study are highlighted (red). AC, Arrhythmogenic right ventricular cardiomyopathy; DCM, Dilated cardiomyopathy; CPVT, Catecholaminergic polymorphic ventricular tachycardia; EDS, Ehlers-Danlos syndrome; FA, Familial arrhythmia; HCM, Hypertrophic cardiomyopathy; LQTS, Long QT syndrome; LVNC, Left ventricular non-compaction cardiomyopathy*.

### Variant Analysis

We selected all variants with a possible effect on protein for analysis (missense, frameshift, stop gained/lost, initiator codon, in-frame insertion, in-frame deletion, and splice-site alterations). Selected variants were further filtered by excluding variants with minor allele frequency > 0.01 among Finnish subjects from general population in dbSNP or The Genome Aggregation Database (gnomAD) database. Pathogenicity of variants were assessed according to American College of Medical Genetics (ACMG) consensus guidelines (9). Benign variants were excluded from the results. Variants that were not classified as benign were further classified as pathogenic/likely pathogenic or as variants of uncertain significance (VUS), based upon the ACMG guidelines by combining data from previous literature, Human Gene Mutation Database (HGMD), ClinVar database, *in silico* prediction tools (SIFT, PolyPhen) and population frequency databases (gnomAD with > 3,000 Finnish controls), The Sequencing Initiative Suomi (SISu) with >10,000 Finnish controls).

### Statistical Analysis

Statistical significance, odds ratios (OR) and 95% confidence intervals (CI) were estimated using χ2 test with two-sided *p*-value (Fisher's Exact Test if a specific variant was detected in multiple study subjects. The Sequencing Initiative Suomi (SISu) database (10), including data on genetic variants from 10,490 exome sequenced Finnish citizens, was used as a control group. Analyses were performed with the Statistical Package for Social Studies version 24.0 (IBM). *P*-values <0.05 were considered statistically significant and all *p*-values were two-sided.

## Results

### Characteristics of the Study Subjects

In the Fingesture study, CAD was determined to be the underlying cause of SCD in 4,392 victims (74.8%), and 3,122 victims (71.1%) had no history of CAD prior to death. Among these victims, single-vessel CAD, determined by evidence of either acute or prior myocardial infarction due to critical stenosis or occlusion of a single epicardial coronary artery, and cardiac hypertrophy were present in 244 individuals. We carried out genetic studies in 95 SCD victims with single-vessel CAD and cardiac hypertrophy in the absence of previously diagnosed CAD, whose DNA passed the quality control for further analysis. No macroscopic or microscopic evidence of cardiomyopathies were observed among these victims. The mean age of the study subjects was 63.6 ± 10.3 years and the majority were men (88.4%, 84 subjects). The mean heart weight was 514.5 ± 87.3 g (range 421-820 g) and the mean BMI 29.0 ± 5.3 kg/m^2^. A healed myocardial infarction scar was detected in 23.2% (22 subjects). 26.3% (25 subjects) had known hypertension, 12.6% (12 subjects) diabetes, 8.4% (8 subjects) dyslipidemia. The most common occluded coronary artery was left anterior descending (84.2% of the subjects; 80 subjects). The characteristics of the study subjects are described in Table 2.

**Table 2:** Clinical characteristics and autopsy findings of the ischemic-SCD victims with single-vessel CAD and cardiac hypertrophy in the absence of previously diagnosed CAD (*n* = 95).

**Characteristic**	**Value**
Age, mean (SD), y	63.6 (10.3)
Male gender, *n* (%)	84 (88.4%)
Hypertension, *n* (%)	25 (26.3%)
Diabetes mellitus, *n* (%)	12 (12.6%)
Dyslipidemia, *n* (%)	8 (8.4%)
Angina, *n* (%)	4 (4.2%)
Dyspnea, *n* (%)	5 (5.3%)
Abundant use of alcohol, *n* (%)	32 (33.7%)
**Circumstances at death**	
Unwitnessed; dead on initial contact, *n* (%)	89 (93.7%)
During physical activity, *n* (%)	5 (5.3%)
In hospital, health center, or ambulance, *n* (%)	3 (3.2%)
Body mass index, mean (SD), kg/m^2^	29.0 (5.3)
Total heart weight, mean (SD), g	514.5 g (87.3), 421-820g range
**Occluded coronary artery**	
LAD, *n* (%)	80 (84.2%)
CX, *n* (%)	4 (4.2%)
RCA, *n* (%)	11 (11.6%)
Myocardial scar, *n* (%)	22 (23.2%)

*CX, Left circumflex coronary artery; LAD, Left anterior descending coronary artery; RCA, Right coronary artery; SD, Standard deviation*.

### Detected Variants

Potentially disease-related variants (pathogenic, likely pathogenic or VUS) were observed in 22 genes among 43 of the study subjects (45.3%), among which pathogenic or likely-pathogenic variants were detected in eight subjects (8.4%). Two or more potentially relevant variants were detected in eight subjects (8.4%).

A summary of the detected pathogenic/likely pathogenic variants is presented in Table 3, the VUS's are presented in the Supplementary Table. Five variants were classified as pathogenic or likely pathogenic and 37 variants were classified as VUS. All of the potentially disease-related variants were detected in myocardial structure protein coding genes, including those associated with arrhythmogenic cardiomyopathy (AC), hypertrophic cardiomyopathy (HCM), dilated cardiomyopathy (DCM) and left ventricular non-compaction cardiomyopathy (LVNC). *RYR2* has also been associated with catecholaminergic polymorphic ventricular tachycardias (CPVTs). None of SCD subjects with rare variants presented autopsy findings specific for classic expression of HCM, DCM, AC or non-compaction cardiomyopathy. No significant variants in ion channel coding genes were observed in the present study. A healed myocardial scar was detected in 10 subjects (23.4%) with potentially disease relevant variant(s). The DNA sequencing data has been deposited to European Variation Archive.

**Table 3:** Summary of pathogenic and likely pathogenic variants in sudden cardiac death victims with single-vessel coronary artery disease and hypertrophied heart found at medico-legal autopsy.

**Mutated gene**	**Nucleotide change**	**Effect of protein**	**Predicted effect**	**N**	**NGS coverage**	**gnomAD > 3,000 Finnish controls MAF**	**SISu > 10,000 Finnish controls MAF**	**ACMG score**	**Clinical features and autopsy findings of the victims**
**Pathogenic variants**									
*DSG2*	c.523 + 1G > A		Affects canonical splicing	1	75	Not detected	<0.0001	PVS1 + PM2 + PP2 + PP5	Male, in his 70 s, BMI 25, heart weight 446 g, no fibrosis, LAD occluded
**Likely pathogenic variants**									
*MYBPC3*	c.2497G > A	p.Ala833Thr	Missense	2	302/363	0.0023	0.0022	PS1 + PP1 + PP2 + PP3	1: Male, in his 50 s, BMI 22, heart weight 450 g, mild fibrosis, LAD occluded 2: Female, in her 70 s, BMI 27, heart weight 598 g, moderate fibrosis, LAD occluded, myocardial scar
*MYH7*	c.3116A > G	p.Glu1039Gly	Missense	3	146/111/101	0.0011	0.0009	PS4 + PM1 + PP2 + PP3	1: Male, in his 60 s, BMI 31, heart weight 540 g, moderate fibrosis, LAD occluded 2: Male, in his 70 s, BMI 28, heart weight 535 g, substantial fibrosis, LAD occluded 3: Male, in his 60 s, BMI 29, heart weight 575 g, mild fibrosis, CX occluded, myocardial scar
*DTNA*	c.362C > T	p.Pro121Leu	Missense	1	96	Not detected	<0.0001	PS3 + PM2 + PP1 + PP4 + PP5	Female, in her 40 s, BMI 28, heart weight 430 g, no fibrosis, LAD occluded
*DSG2*	c.166G > A	p.Val56Met	Missense	1	129	0.0006	0.0004	PM1 + PP2 + PP3 + PP4 + PP5	Male, in his 50 s, BMI 31, heart weight 580 g, moderate fibrosis, LAD occluded, myocardial scar

*ACMG criteria:*

*Very strong evidence of pathogenicity: PVS1, Null variant (nonsense, frameshift, canonical ±1 or 2 splice sites, initiation codon, single or multi-exon deletion) in a gene where loss of function is a known mechanism of disease*.

*Strong evidence of pathogenicity: PS1, Same amino acid change as a previously established pathogenic variant regardless of nucleotide change; PS2, De novo mutation in a patient with the disease and no family history; PS3, Well-established in vitro or in vivo functional studies supportive of a damaging effect on the gene or gene product; PS4, The prevalence of the variant in affected individuals is significantly increased compared to the prevalence in controls*.

*Moderate evidence of pathogenicity: PM1, Located in a mutational hot spot and/or critical and well-established*.

*Functional domain (e.g., active site of an enzyme) without benign variation; PM2, Absent from controls (or at extremely low frequency if recessive)*.

*In exome sequencing project, 1,000 Genomes or gnomAD; PM3, For recessive disorders, detected in trans with a pathogenic variant; PM4, Protein length changes due to in-frame deletions/insertions in a non-repeat region or stop-loss variants; PM5, Novel missense change at an amino acid residue where a different missense change determined to be pathogenic has been seen before; PM6, Assumed de novo, but without confirmation of paternity and maternity*.

*Supporting evidence of pathogenicity: PP1, Co-segregation with disease in multiple affected family members in a gene definitively known to cause the disease; PP2, Missense variant in a gene that has a low rate of benign missense variation and where missense variants are a common mechanism of disease; PP3, Multiple lines of computational evidence support a deleterious effect on the gene or gene product (conservation, evolutionary, splicing impact, etc.); PP4, Patient's phenotype or family history is highly specific for a disease with a single genetic etiology; PP5, Reputable source recently reports variant as pathogenic but the evidence is not available to the laboratory to perform an independent evaluation*.

*Rules for combining criteria to classify sequence variants*.

*Pathogenic:*

*1. 1 Very Strong (PVS1) AND*.

*a. ≥1 Strong (PS1–PS4) OR*.

*b. ≥2 Moderate (PM1–PM6) OR*.

*c. 1 Moderate (PM1–PM6) and 1 Supporting (PP1–PP5) OR*.

*d. ≥2 Supporting (PP1–PP5)*.

*2. ≥2 Strong (PS1–PS4) OR*.

*3. 1 Strong (PS1–PS4) AND*.

*a. ≥3 Moderate (PM1–PM6) OR*.

*b. 2 Moderate (PM1–PM6) AND ≥2 Supporting (PP1–PP5) OR*.

*c. 1 Moderate (PM1–PM6) AND ≥4 Supporting (PP1–PP5)*.

*Likely pathogenic:*

*1. 1 Very Strong (PVS1) AND 1 Moderate (PM1–PM6) OR*.

*2. 1 Strong (PS1–PS4) AND 1–2 Moderate (PM1–PM6) OR*.

*3. 1 Strong (PS1–PS4) AND ≥ 2 Supporting (PP1–PP5) OR*.

*4. ≥3 Moderate (PM1–PM6) OR*.

*5. 2 Moderate (PM1–PM6) AND ≥ 2 Supporting (PP1–PP5) OR*.

*6. 1 Moderate (PM1–PM6) AND ≥ 4 Supporting (PP1–PP5)*.

*ACMG, American college of molecular genetics; BMI, Body mass index; CX, Left circumflex coronary artery; gnomAD, The Genome Aggregation Database; LAD, Left anterior descending coronary artery; MAF, Minor allele frequency; NGS, Next generation sequencing; SISu, The Sequencing Initiative Suomi*.

### Pathogenic and Likely-Pathogenic Variants

A novel pathogenic variant was found in the desmosomal gene *DSG2*, of which mutations have been described in patients with AC. This variant (c.523 + 1G > A) was considered as pathogenic because of its effect on splicing site, low population frequency in Finland (<1/10,000) and classification as likely pathogenic by ClinVar database. Additionally, in a previous study (11), similar variant (c.523 + 2T > C) was found in three unrelated patients with AC.

Four likely pathogenic missense variants were identified among the study subjects in *MYBPC3* (two subjects), *MYH7* (three subjects), *DTNA* and *DSG2*. These variants did not quite fulfill the criteria to be considered as pathogenic. However, these variants had low minor population frequency in Finland, are considered as disease causing by ClinVar, and/or are predicted to be damaging by *in silico* tools. The Ala833Thr variant in *MYBPC3* associated with HCM was detected in two unrelated subjects. It was also classified as likely-pathogenic in a previous study (12), and found in familial HCM in a Swedish study (13). Compared to Finnish control population, the difference in the prevalence of affected carriers was statistically significant (2/95 vs. 46/10,480; *p* = 0.016, OR = 4.9, 95% CI 1.2-20.4). The p.Val56Met variant in a conserved region of *DSG2* was previously observed in an AC patient (14), and p.Pro121Leu in *DTNA* has been previously described in family with left ventricular non-compaction (15). We also observed a p.Glu1039Gly variant in a conserved region of *MYH7* in three victims. This variant was previously detected in SCD victims with primary myocardial fibrosis (PMF) (12), and a mutation next to this codon in highly conserved region (p.Leu1038Pro) was previously associated with DCM (16). The difference in the prevalence of affected carriers compared to control population was statistically significant (3/95 vs. 18/10,489; *p* = 0.0008, OR = 19.0, 95% CI 5.5-65.5).

### Variants of Uncertain Significance

Variants classified as VUS were observed in 39 study subjects (41.1%). Five of the VUSs were detected in multiple unrelated affected subjects, suggesting possible pathogenicity. These included p.Thr358Ile in *DSC2* (*n* = 3), p.Lys259Glu in *TPM1* (*n* = 3), p.Arg100His in *CSRP3* (*n* = 2), p.Ala936Val in *MYH6* (*n* = 2) and p.Arg634His in *DSC2* (*n* = 4). Previously, p.Arg100His in *CSRP3* has been found in Danish HCM patient (17). p.Ala292Ser in *CASQ2* and p.Arg31Gln in *DTNA* have both been detected in PMF victims (12). p.Ser140Phe in *PKP2* has been described in patients with AC (18, 19), as well as in a previous DCM study (20), which considered this variant as *at least disease modifying if not pathogenic*. p.Thr1373Ala in conserved region of *DSP* was classified earlier as potentially disease causing in a Finnish AC study (21). This study also showed some evidence for a role of the variant in risk of tachycardias and association of both PR-interval prolongation and abnormality in the atrioventricular conduction. p.Thr358Ile in *DSC2* was found in three study subjects and earlier in a patient with AC (22). p.Ala2499Thr, p.Arg3597Gly and p.Asp1862Ala in *RYR2* has not been described in previous literature, but p.Arg2518Trp was observed in PMF victim (12). p.Gly154Ser in *CRYAB* was previously classified as likely pathogenic (12), and observed in DCM patients (23). However, the evidence of pathogenicity remains unclear. p.Ala2294Gly in *DSP* has previously been classified as pathogenic (24). The mutation was found in two patients with advanced DCM undergoing cardiac transplantation and the mutation also co-segregated with DCM in the other family. p.Arg1037Gln in *LAMA4* have previously been described in the PMF study (12).

## Discussion

In the present study, derived from our large autopsy-based SCD population, we investigated the genetic background for LV hypertrophy, with or without fibrosis, among ischemic SCD victims with single vessel CAD found at autopsy. Our genetic studies demonstrated that many CAD-associated SCD victims with single vessel disease and myocardial hypertrophy carry rare variants in cardiomyopathy-associated genes without anatomical or histopathological findings of the inherited cardiomyopathies. Likely disease-causing gene variants were detected in 8.4% of the subjects and 41.1% carried a variant of uncertain significance. All rare variants were detected predominantly in myocardial structure coding genes associated with DCM, AC, HCM, and LVNC. However, mutations in the *RYR2* gene have been associated with both catecholaminergic polymorphic ventricular tachycardias, in addition to AC (25). No significant variants in ion channel coding genes were detected among our study subjects.

These results raise a question whether, at least in some ischemic SCD victims, variants in myocardial structural genes may possibly contribute to the development of myocardial hypertrophy and/or fibrosis and correlate with the risk for fatal arrhythmias, especially when the severity of CAD does not appear to entirely explain the cause of SCD. However, we cannot exclude the possibility of myocardial hypertrophy with/without fibrosis being only a bystander in specific cases in which an acute coronary event leading to SCD. In addition, about 23% of the study subjects had a myocardial scar detected at autopsy. Healed scar can induce myocardial hypertrophy adjacent to the scar (26), that may associate with risk of arrhythmias, while a genetic predisposition to hypertrophy and fibrosis may associate with these findings in remote, non-infarcted areas, further affecting the vulnerability to arrhythmias. The causes of hypertrophy and fibrosis in the study subjects are likely to be multifactorial, including not only ischemic modulation, but also different levels of treated or untreated hypertension. Nevertheless, it remains interesting that many subjects also have the possibly myocardial disease-causing genetic variants. Perhaps the current approach of labeling each cardiac disease as its own entity is too narrow a view of the actual pathophysiology underlying the risk for sudden death.

Likely disease-causing variants in the present study were detected in genes associated with AC, HCM, DCM, and LVNC. However, none of the subjects in our study presented characteristic autopsy findings related to these specific cardiomyopathies. Rather, all deaths were determined to be caused by CAD in medicolegal investigations, in the presence of LV hypertrophy with varying degrees of fibrosis. As previously observed (12, 27, 28) the phenotypic expression of specific inherited cardiomyopathies may vary significantly. Fibrosis may be the only, or perhaps the earliest, expression of the underlying structural disorder. Inherited structural disorders may have overlapping features (12), which were seen in our results as well. In general, several variants were found in some of the same genes as in PMF victims (12).

Phenotypic expressions and the age at onset of the myocardial disease between gene variants are very heterogenous. For example, variants in *MYBPC3* have been associated with late onset disease and a high rate of incomplete penetrance (29, 30). Even within the same gene, the disease severity and penetrance rate of different gene defects and variants can vary significantly (31). Variable phenotypes can occur also in individuals with the same disease-causing mutation. Although no autopsy findings relating to particular cardiomyopathies were found in any of the study victims, it is reasonable to suggest that the gene defect(s) might have a role in the development of cardiac hypertrophy.

Cardiac hypertrophy is generally known to be associated with increased risk for fatal arrhythmias and SCD (4, 32), and as observed in subjects with CAD (33), it may have an independent mechanistic pathway for ventricular arrhythmias (32). For example, increased left ventricular mass impairs the coronary flow and increases oxygen demand, which can lead to ischemia and arrhythmias, even in the presence of less severe CAD (34). Thus, the risk for SCD was likely higher than with single-vessel disease in the absence of hypertrophy/fibrosis in our study. Almost one-half of the study subjects carried a variant in myocardial structural coding genes, which might have, at least in some cases, explained the cardiac hypertrophy. Even though the panel we used included 174 myocardial genes, we cannot exclude the possibility that some of the study subjects might have had unidentified in other genes.

The number of variants classified as VUS were rather high in the present study. The development of NGS techniques has provided a relatively fast and affordable way for sequencing of large gene panels, which has led to increasing amount of genetic testing and studies. Thus, the number of variants classified as VUS has increased, which is a common challenge in clinical genomics (35). ACMG guidelines are widely used for interpretation and reporting of gene variants. However, since guidelines have not been validated for specific disorders, they may be suboptimal for some genetic diseases like CPVT (36). Even if a genetic variant is not considered pathogenic, it may still be reasonable to consider the possibility of disease-modifying role and contributing effect on the risk of SCD, especially in the presence of CAD.

In cardiomyopathies, the abnormal myocardial structure is considered to act as the substrate for arrhythmias. It has been observed that arrhythmias may occur in the early phase of the disease process or even in the absence of phenotypic features associated with cardiomyopathies, suggesting that underlying gene defects may have also other arrhythmogenic mechanisms (37). In our CAD-SCD victims, the risk profile for SCD might be a combination of components from both CAD and structural gene defects.

The severity of inherited cardiac diseases has been shown to increase with double or compound mutations (31, 38). Among our study population, 8.4% of the study subjects had at least two potentially relevant variants in structural genes. It is reasonable to consider that individuals with multiple variants might have higher risk for the fatal arrhythmias. Also, it might be possible that even if subject does not have likely disease-causing variant, but multiple VUS variants, the risk for arrhythmias may be higher.

While traditional risk factors for SCD and CAD have been widely studied, the importance and limitations of personalized risk prediction and the role of genetics in the identification of individuals at high risk for SCD has been recognized (39). Evidence for the role of heredity in the risk of SCD have been demonstrated (4), and it is anticipated that genetic profiling will have an increasingly important role in the assessment of the arrhythmogenic risk in the future (37, 39). Based upon the results of this study, we suggest that gene variants in myocardial structure coding genes may associate with the risk of SCD in the presence of single vessel CAD, which would support more intensive CAD treatment strategies for such patients. Additionally, the findings enhance the necessity of primary prevention of CAD (e.g., a healthy lifestyle, cholesterol and blood pressure control), particularly among individuals with variants in myocardial structure coding genes. Therefore, it could be relevant to screen the families of victims with the profiles observed in this report in order to identify those at risk for SCD and to minimize their cardiovascular disease risk burden.

Postmortem genetic analysis improves the accuracy of determining the cause of death, especially when a structurally normal heart is detected at autopsy or when the severity of heart disease does not entirely explain the cause of sudden death (40). Generally, when CAD is detected at autopsy, especially in middle-aged and older populations, postmortem genetic analysis is not considered as necessary to identify the cause of death. These results suggest that rare variants in myocardial structure coding genes might have a contributing role in the development of myocardial disease among patients with less severe coronary artery disease, however, the usefulness in diagnostics, for example, is yet to be solved.

## Limitations

Since our NGS method used is unable of identifying, for example, mutations in intron regions, large deletions or copy number variations, potential variants may go undiscovered. As in most NGS studies, the causal association between detected rare variants and cardiac structural disease remains to be established. Multiple variants with uncertain pathogenicity were observed in the present study, and thus more studies are needed to clarify the specific role of these mutations in the pathophysiology of SCDs. Since none of the subjects in our study had prior knowledge of cardiac disease, it is possible that they had underlying hypertension that could explain the occurrence of LVH. However, this only increases the possibility that, in subjects without prior hypertension, the occurrence and relevance of underlying genetic variants could be even greater. Also, well-recognized limitations are present when extracting DNA from formalin fixed paraffin embedded tissues. However, NGS coverage in all cases were sufficient and Sanger confirmation was not considered necessary.

## Conclusions

Many SCD victims with single vessel CAD and LV hypertrophy observed at autopsy, in the absence of previously diagnosed CAD, had rare variants in myocardial structural coding genes. Variants were detected in disease-associated genes including HCM, DCM, AC, LVNC and CPVT. We identified no variants in genes that are exclusively associated with inherited ion channel defects among the study subjects. The variants identified might contribute to the risk for fatal arrhythmias and sudden death in ischemic heart disease, in SCD victims with less advanced CAD.

## Data Availability Statement

The datasets presented in this study can be found in online repositories. The names of the repository/repositories and accession number(s) can be found at: https://www.ebi.ac.uk/eva/; PRJEB47695, ERZ3584884.

## Ethics Statement

This study complies with the Declaration of Helsinki and has been approved by the Ethics Committee of the University of Oulu and Finland’s Ministry of Social Affairs and Health. Finnish Institute for Health and Welfare and the Regional State Administrative Agency of Northern Finland approved the review of autopsy data by the investigators. Written informed consent for participation was not required for this study in accordance with the national legislation and the institutional requirements.

## Author Contributions

JJ, HH, and JP: study design and conception. JV and JJ: drafting of the manuscript and obtained funding. JV and LH: statistical analysis. LH, KPy, SS, KPo, LP, KK, JP, RK, RM, HH, and JJ: critical revision of the manuscript for important intellectual content. JJ and HH: supervision. All authors contributed to the article, acquisition, analysis or interpretation of data, and approved the submitted version.

## Funding

This work was supported by Aarne Koskelo Foundation, Finnish Foundation for Cardiovascular Research, The Finnish Medical Foundation, Instrumentarium Science Foundation, The Maud Kuistila Memorial Foundation, Ida Montin Foundation, The University of Oulu Scholarship Foundation, Paavo Nurmi Foundation, and Maire Taponen Foundation.

## Conflict of Interest

The authors declare that the research was conducted in the absence of any commercial or financial relationships that could be construed as a potential conflict of interest.

## Publisher's Note

All claims expressed in this article are solely those of the authors and do not necessarily represent those of their affiliated organizations, or those of the publisher, the editors and the reviewers. Any product that may be evaluated in this article, or claim that may be made by its manufacturer, is not guaranteed or endorsed by the publisher.
