# Supplementary material for: Genetic Variants Associated With Sudden Cardiac Death in Victims With Single Vessel Coronary Artery Disease and Left Ventricular Hypertrophy With or Without Fibrosis
Source: Front Cardiovasc Med. 2022 Jan 11;8:755062. doi: 10.3389/fcvm.2021.755062 (PMC8788946; doi:10.3389/fcvm.2021.755062)
Supplement: Supplementary file 1 [file Table_1.DOCX]

**Supplement material.** Summary of variants of uncertain significance in sudden cardiac death victims with single-vessel coronary artery disease and hypertrophied heart found at medico-legal autopsy. *gnomAD = The Genome Aggregation Database, MAF =Minor allele frequency, NGS = Next generation sequencing, SISu = The Sequencing Initiative Suomi.*

| Mutated Gene | Nucleotide Change | Effect of Protein | Predicted Effect | N | NGS Coverage | gnomAD >3,000 Finnish controls MAF | SISu>10,000 Finnish controls MAF |
| --- | --- | --- | --- | --- | --- | --- | --- |
| *CASQ2* | c.874G>T | Ala292Ser | Missense | 1 | 392 | 0.0020 | 0.0023 |
| *DSC2* | c.1073C>T | Thr358Ile | Missense | 3 | 250/118/113 | 0.0035 | 0.0040 |
| *MYH6* | c.3010G>T | Ala1004Ser | Missense | 1 | 114 | 0.0006 | 0.0006 |
| *DSP* | c.3600T>G | Asn1200Lys | Missense | 1 | 152 | 0.0001 | <0.0001 |
| *RYR2* | c.7495G>A | Ala2499Thr | Missense | 1 | 126 | 0.0012 | 0.0007 |
| *PKP2* | c.419C>T | Ser140Phe | Missense | 1 | 217 | 0.0029 | 0.0014 |
| *ANKRD1* | c.417C>A | Phe139Leu | Missense | 1 | 200 | 0.0048 | 0.0051 |
| *MYH6* | c.1261G>A | Val421Met | Missense | 1 | 145 | 0.0009 | 0.0006 |
| *DTNA* | c.92G>A | Arg31Gln | Missense | 1 | 113 | 0.0055 | 0.0057 |
| *MYH6* | c.2807C>T | Ala936Val | Missense | 2 | 156/111 | 0.0047 | 0.0053 |
| *DSP* | c.4117A>G | Thr1373Ala | Missense | 1 | 297 | 0.0001 | <0.0001 |
| *CSRP3* | c.299G>A | Arg100His | Missense | 2 | 86/255 | 0.0053 | 0.0047 |
| *TTN* | c.34150_34170delGTTCTA­CCTGAAGAAGAGGAA | Val11384_Glu11390del | Inframe deletion | 1 | 642 | 0.0001 | <0.0001 |
| *LDB3* | c.566C>T | Ser189Leu | Missense | 1 | 266 | 0.0001 | 0.0009 |
| *TPM1* | c.775A>G | Lys259Glu | Missense | 3 | 116/176/74 | 0.0045 | 0.0054 |
| *DTNA* | c.1693C>T | Pro565Ser | Missense | 1 | 103 | Not detected | Not detected |
| *RYR2* | c.10789A>G | Arg3597Gly | Missense | 1 | 13 | Not detected | Not detected |
| *RYR2* | c.9190G>A | Ala3064Thr | Missense | 1 | 60 | Not detected | 0.0008 |
| *DSP* | c.6881C>G | Ala2294Gly | Missense | 1 | 112 | 0.0003 | 0.0002 |
| *RYR2* | c.5585A>C | Asp1862Ala | Missense | 1 | 112 | Not detected | Not detected |
| *JUP* | c.1996G>A | Val666Met | Missense | 1 | 180 | Not detected | Not detected |
| *NEXN* | c.968A>G | Glu323Gly | Missense | 1 | 41 | 0.0021 | 0.0014 |
| \| ***ACTN2*** \| \| --- \| | c.1297T>G | Ser433Ala | Missense | 1 | 123 | Not detected | Not detected |
| *DSP* | c.4558A>G | Ser1520Gly | Missense | 1 | 79 | Not detected | Not detected |
| *LAMA4* | c.3110G>A | Arg1037Gln | Missense | 1 | 39 | 0.0015 | 0.0021 |
| *RYR2* | c.7552C>T | Arg2518Trp | Missense | 1 | 231 | Not detected | 0.0003 |
| *LAMA4* | c.3054G>T | Leu1018Phe | Missense | 1 | 152 | 0.0027 | 0.0021 |
| *NEXN* | c.1539G>A | Met513Ile | Missense | 1 | 13 | Not detected | Not detected |
| *MYLK2* | c.902C>A | Thr301Asn | Missense | 1 | 123 | 0.0022 | 0.0032 |
| *DSP* | c.7213C>A | Leu2405Ile | Missense | 1 | 12 | Not detected | Not detected |
| *MYBPC3* | c.2870C>G | Thr957Ser | Missense | 1 | 168 | 0.0003 | 0.0002 |
| *DSP* | c.1696G>A | Ala566Thr | Missense | 1 | 28 | 0.0004 | 0.0003 |
| *ABCC9* | c.4238G>T | Cys1413Phe | Missense | 1 | 13 | Not detected | Not detected |
| *PKP2* | c.1460A>G | Gln487Arg | Missense | 1 | 39 | Not detected | Not detected |
| *CRYAB* | c.460G>A | Gly154Ser | Missense | 1 | 58 | 0.0007 | 0.0016 |
| *DSC2* | c.1901G>A | Arg634His | Missense | 4 | 87/86/107/38 | 0.0053 | Not detected |
| *LAMA4* | c.3283-3C>G |  | Affects canonical splicing | 1 | 67 | 0.0003 | 0.0002 |
